# Heterogeneous Nucleation of Protein Crystals on Fluorinated Layered Silicate

**DOI:** 10.1371/journal.pone.0022582

**Published:** 2011-07-27

**Authors:** Keita Ino, Itsumi Udagawa, Kazuki Iwabata, Yoichi Takakusagi, Munehiro Kubota, Keiichi Kurosaka, Kazuhito Arai, Yasutaka Seki, Masaya Nogawa, Tatsuo Tsunoda, Fujio Mizukami, Hayao Taguchi, Kengo Sakaguchi

**Affiliations:** 1 Department of Applied Biological Science, Tokyo University of Science, Noda, Chiba, Japan; 2 Iwaki Lab, Kunimine Industries Co., Ltd., Joban Iwaki, Fukushima, Japan; 3 Research Center for Compact Chemical System, National Institute of Advanced Industrial Science and Technology, Tsukuba, Ibaraki, Japan; Massachusetts Institute of Technology, United States of America

## Abstract

Here, we describe an improved system for protein crystallization based on heterogeneous nucleation using fluorinated layered silicate. In addition, we also investigated the mechanism of nucleation on the silicate surface. Crystallization of lysozyme using silicates with different chemical compositions indicated that fluorosilicates promoted nucleation whereas the silicates without fluorine did not. The use of synthesized saponites for lysozyme crystallization confirmed that the substitution of hydroxyl groups contained in the lamellae structure for fluorine atoms is responsible for the nucleation-inducing property of the nucleant. Crystallization of twelve proteins with a wide range of pI values revealed that the nucleation promoting effect of the saponites tended to increase with increased substitution rate. Furthermore, the saponite with the highest fluorine content promoted nucleation in all the test proteins regardless of their overall net charge. Adsorption experiments of proteins on the saponites confirmed that the density of adsorbed molecules increased according to the substitution rate, thereby explaining the heterogeneous nucleation on the silicate surface.

## Introduction

The rapid development of new technologies in the area of structural biology has significantly increased our understanding of the structure-function relationship of proteins. One difficulty associated with increasing the throughput in X-ray crystallography of proteins is their poor crystallizability. Appropriate conditions for crystallization of a new protein are usually determined by a screening procedure, which includes multi-dimensional trials on various solution variables. Such trials are time-consuming and may lead to either no crystal formation or crystals that are unsuitable for structural investigations. Problems such as these arise because homogeneous nucleation of protein crystals generally requires specific and restricted supersaturation [1], [2]. Hence, there is requirement for an improved screening system that can control the nucleation process [3], [4].

Several approaches have been developed to improve the screening procedure based on heterogeneous nucleation mediated by a nucleant. Since the pioneering experiments of McPherson and Shlichta, who tested 50 different minerals as potential nucleants for crystallization, several different types of nucleant have been developed [5]–[33]. Some studies describe the use of charged surfaces, including chemically modified mica [6]–[8], poly-L-lysine surface [9], [10], polymeric film [11] and patterned silicon [12], [13], as potential nucleants. These procedures rely on electrostatic interactions between the charged surface and the protein with a net charge of the opposite sign. In this way, the surface seems to promote nucleation of certain proteins. Several studies have shown porous media such as porous silicon [14]–[16], bioactive gel-glass [17] and carbon nanotube based materials [18] act as effective nucleants for the crystallization of proteins. The pores promote aggregation of proteins such that a spontaneous critical nucleus comprised of tens of protein molecules form inside each pore. Another study with respect to a porous material has shown that zeolites with regular micropores induce heteroepitactic nucleation [19]. A common feature of these nucleants is that their specific surface properties govern the nucleation potential of the proteins.

Our previous study has shown that two layered silicates, a K-tetrasilicic fluoromica with a 2∶1 layer comprised of tetrahedral and octahedral units and a chlorite with a 2∶1∶1 layer, act as nucleants for crystallization of model proteins [20]. The fluoromica promoted nucleation in eight out of the ten proteins tested. By contrast, the chlorite surface promoted nucleation in only two out of the ten test proteins. Not much is known about the factors that contribute to the nucleation potential of silicates. Thus, it was difficult to achieve an efficient and reliable screening system for the crystallization of a variety of proteins in a controlled manner using conventional silicates.

Layered silicate is a phyllosilicate having a parallel two-dimensional lamellar structure as shown in Fig. S1 [34]. The tetrahedral layer at least consists of tetrahedral silicon-oxygen bonds linked together. The apical oxygen of the layer points in the same direction as the coplanar base of the silicon-oxygen network. The octahedral layer consists of octahedral magnesium-oxygen and/or aluminum-oxygen bonds linked together and lie between two tetrahedral layers pointing toward each other to share their oxygen. The octahedral corners not filling in the plane of apical oxygen are occupied by hydroxyl group or fluorine atom, which possess similar ionic radii to each other [34]–[37]. In terms of chemical composition, the fluoromica contains fluorine atoms, whereas the chlorite contains hydroxyl groups and lacks any fluorine atoms. These features suggest that the fluorine atoms contained in the lamellae network might be an important factor affecting heterogeneous nucleation.

This article relates to the use of fluorinated layered silicate as a nucleant for protein crystallization. Our results reveal the role of the fluorine atoms in heterogeneous nucleation of model proteins.

## Results and Discussion

### Lysozyme crystallization using layered silicates with different chemical compositions

**Figure 1:**
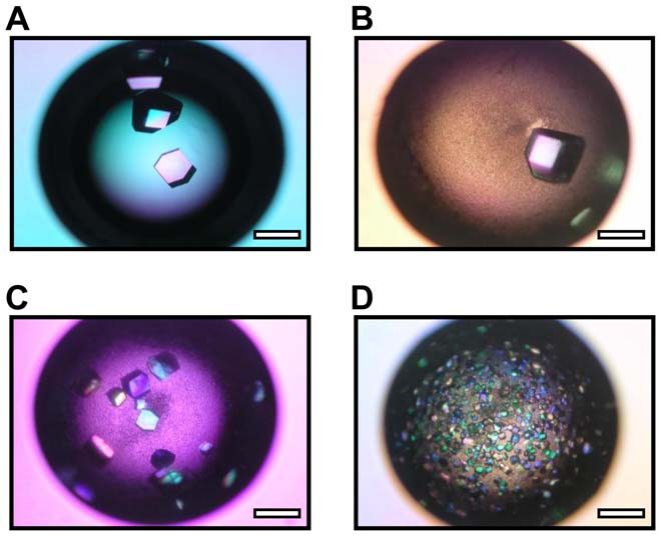
Morphologies of lysozyme crystals in the presence of layered silicates. Micrographs of lysozyme crystals in the absence of layered silicate (Control) (A) and in the presence of sericite (B), K-tetrasilicic fluoromica (C) and Na-tetrasilicic fluoromica (D). Crystallization conditions: starting lysozyme concentration 20 mg/mL, starting silicate content 0.2%, precipitant agent sodium chloride 1.0 M in sodium acetate 0.2 M, pH 4.7. Scale bar: 0.5 mm.

**Figure 2:**
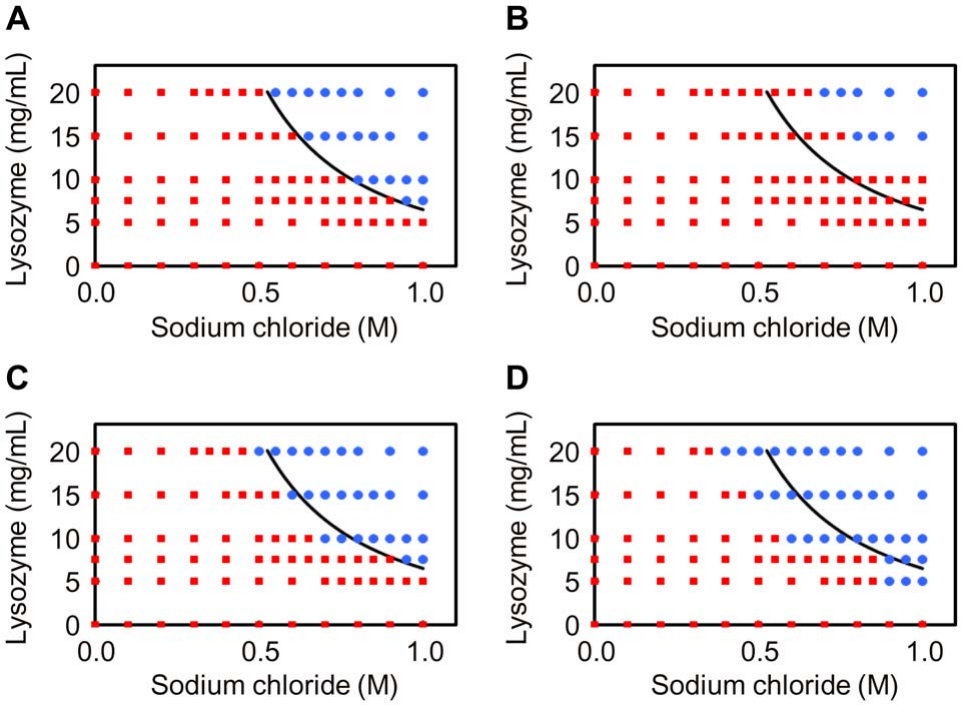
Concentration range for precipitating lysozyme crystals in the presence of layered silicates. Crystallization phase diagrams for lysozyme in the absence of layered silicate (Control) (A) and in the presence of sericite (B), K-tetrasilicic fluoromica (C) and Na-tetrasilicic fluoromica (D). The variables are starting concentrations of lysozyme and sodium chloride. The blue circles and the red squares represent conditions at which crystallization does or does not occur, respectively. The curve indicates the supersolubility curve separating conditions where crystallization occurs from those in which the crystallization solution remains clear in the absence of silicate.

Eleven silicates with 2∶1 type lamellar structure and different chemical compositions were used as potential heterogeneous nucleants for lysozyme crystallization. Four (fluorophlogopite, fluorohectorite, K-tetrasilicic fluoromica and Na-tetrasilicic fluoromica) of the eleven silicates include fluorine, which were identified by SEM-EDX analyses. We crystallized lysozyme by the hanging-drop vapor diffusion technique in the absence and presence of the silicates. Lysozyme is an ellipsoidal protein whose dimensions are 4.5×3.0×3.0 nm and with a molecular weight of 14.3 kDa. For a low price is easy to obtain commercially a high purity sample. In addition, it is well known its crystallization conditions, which allows testing the adsorption capacities and kinetics on inorganic materials [38], [39] as well as the nucleation potential of materials. As shown in Table 1, the four fluorosilicates decreased the crystallization time and the crystal size but increased the nucleation density by comparison with the control samples. These findings suggest that fluorosilicates promote nucleation of the lysozyme crystal. By contrast, the remaining seven silicates that lack fluorine atoms (Table 1) promoted an increase in both the crystallization time and the crystal size and a decrease in the crystal density, suggesting that these surfaces suppress nucleation. No significant differences in the crystal morphology were observed both in the absence (Fig. 1A) and presence of silicate (Fig. 1B–D), indicating that the silicates do not influence the crystal symmetry. The crystallization phase diagrams in the presence of sericite showed that lysozyme crystallization did not take place in some conditions where spontaneous crystallization occurs in the absence of silicate (Fig. 2A and 2B). By contrast, the concentration range for precipitating crystals expanded in the presence of the fluoromicas compared with the control experiment (Fig. 2A, C and D). These observations indicate that the fluoromicas form stable nuclei heterogeneously at lower supersaturation conditions than required for homogeneous nucleation. Our findings showed a correlation between the presence of constituent fluorine atoms and the action of the silicates. The fluorine atoms are located on the terminals of the lamella structure at the same position normally occupied by hydroxyl groups in the case of silicates lacking fluorine atoms [34]–[37]. Therefore, these experiments indicated that the heterogeneous nucleation phenomenon is governed by the substitution of the hydroxyl groups for fluorine atoms.

**Table 1:** Crystallization times, crystal sizes and nucleation densities for lysozyme in the presence of layered silicates.

Layered silicate		Time	Size	Density
Sample name	Idealized structural formula	(day)	(mm)	(n.c./mm^2^)
Control	-	2	0.44±0.11	0.49±0.31
Pyrophyllite	Al_2_Si_4_O_10_(OH)_2_	6	0.62±0.15	0.30±0.16
Hectorite	Na_0.33_Mg_2.67_Li_0.33_Si_4_O_10_(OH)_2_	5	0.78±0.09	0.15±0.04
Talc	Mg_3_Si_4_O_10_(OH)_2_	4	0.82±0.09	0.15±0.02
Muscovite	KAl_3_Si_3_O_10_(OH)_2_	4	0.74±0.14	0.17±0.07
Saponite	Na_0.33_Mg_3_Al_0.33_Si_3.67_O_10_(OH)_2_	4	0.52±0.14	0.34±0.19
Montmorillonite	Na_0.33_Mg_0.33_Al_1.67_Si_4_O_10_(OH)_2_	3	0.72±0.14	0.21±0.14
Sericite	KAl_3_Si_3_O_10_(OH)_2_	3	0.71±0.13	0.21±0.07
Fluorophlogopite	KMg_3_AlSi_3_O_10_F_2_	1.5	0.30±0.12	1.71±1.11
Fluorohectorite	Na_0.33_Mg_2.67_Li_0.33_Si_4_O_10_F_2_	1.5	0.29±0.12	2.14±1.33
K-tetrasilicic fluoromica	KMg_2.5_Si_4_O_10_F_2_	1.5	0.28±0.08	2.72±1.52
Na-tetrasilicic fluoromica	NaMg_2.5_Si_4_O_10_F_2_	1.3	0.08±0.03	25.0±12.4

Crystallization conditions: starting lysozyme concentration 20 mg/mL, starting silicate content 0.2%, precipitant agent sodium chloride 1.0 M in sodium acetate 0.2 M, pH 4.7. Crystallization time was defined as the time that elapses before the appearance of the first crystal. Crystal size refers to the average length of the longitudinal axis of the crystal calculated on a set of several hundreds of crystals. Nucleation density is expressed as the number of crystals per mm^2^. Reported values are mean ± SD, *n*>40.

### Characterization of synthesized fluorinated saponites

Three fluoro-substituted saponites (F-Saps) were analyzed by XRD (Fig. S2). The presence of a (060) reflection at 1.52 nm in the diffraction patterns revealed that the synthesis procedure resulted in a trioctahedral clay-like structure. The absence of a peak at 39° in the diffraction patterns indicates the lack of free NaF in the synthesized products. XPS analyses showed that the fluorine content of F_0_-Sap, F_0.114_-Sap and F_0.188_-Sap were 0.00, 0.53 and 0.86%, respectively (Table S1), giving a structural formulae of Na_0.178_Mg_2.468_Al_0.425_Si_3.903_O_10_(OH)_2_, Na_0.251_Mg_2.531_Al_0.435_Si_3.846_O_10_F_0.114_(OH)_1.886_ and Na_0.273_Mg_2.531_Al_0.427_Si_3.846_O_10_F_0.188_(OH)_1.812_, respectively. Note that here we abbreviate each saponite as F_x_-Sap, where x corresponds to the substitution rate. A study of nitrogen adsorption/desorption showed that the isotherms of F-Saps were typical for layered materials corresponding to type IV in IUPAC classification, with type H2 hysteresis loop at relative pressures between 0.40 and 1.00 (Fig. S3). The observed shape of this hysteresis loop is indicative of the existence of mesopores. Total pore volume of F_0_-Sap, F_0.114_-Sap and F_0.188_-Sap were 0.18, 0.14 and 0.12 cm^3^/g, respectively. These mesoporosities derive from the house-of-card structure, which is the disordered stacking of nanolayers disposed edge-to-face [37], [40], [41]. The specific surface area of F-Saps decreased with the substitution rate. This result was anticipated because of the increase in crystallinity observed in the XRD patterns (Table S2 and Fig. S2). The CEC, often used as a measure of the layer charge [42], increased as the substitution rate increased (Table S2). Furthermore, the zeta potential in 0.2 M sodium acetate (pH 4.7) containing 1.0 M sodium chloride fell with the substitution rate (Fig. S4). No significant change in the hydrophobicity was observed in F-Saps (Fig. S4). These measurements indicate that substitution increases the surface charge density of the layer. Because the surface of F-Sap is highly negatively charged it attracts lysozyme molecules *via* electrostatic interactions under suitable crystallization conditions.

Fluoro-alkylated saponites (FA-Saps) were analyzed by FT-IR spectroscopy (Fig. S5). The peaks observed at 1000–1100 cm^−1^ correspond to the saponite lattice vibrations. The peaks of around 1200 cm^−1^ correspond to C–F stretching vibration in the -CF_2_- and -CF_3_ groups, which increased in intensity with increasing levels of silane treatment. This observation indicates that the fluoroalkyl chains have been introduced into the saponite. We abbreviate each saponite as FA_x_-Sap, where x corresponds to the coverage.

### Lysozyme crystallization using synthesized fluorinated saponites

**Figure 3:**
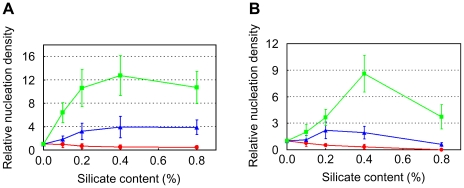
Relative nucleation densities for lysozyme in the presence of F-Saps. Red circle: F_0_-Sap, blue triangle: F_0.114_-Sap, green square: F_0.188_-Sap. (A) Starting lysozyme concentration: 20 mg/mL. Reported values are mean ± SD, *n* = 6. (B) Starting lysozyme concentration: 15 mg/mL. Reported values are mean ± SD, *n* = 6.

**Figure 4:**
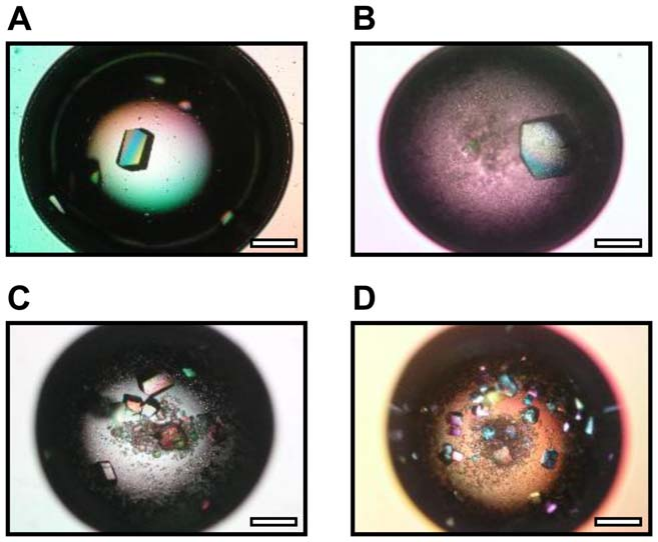
Morphology of lysozyme crystals in the presence of F-Saps. Micrographs of lysozyme crystals in the absence of layered silicate (Control) (A) and in the presence of F_0_-Sap (B), F_0.114_-Sap (C) and F_0.188_-Sap (D). Crystallization conditions: starting lysozyme concentration 20 mg/mL, starting silicate content 0.2%, precipitant agent sodium chloride 1.0 M in sodium acetate 0.2 M, pH 4.7. Scale bar: 0.5 mm.

To investigate whether the substitution of hydroxyl groups for fluorine atoms governs the heterogeneous nucleation, we analyzed the crystallization of the model protein lysozyme on the surface of F-Saps. By comparison with the control experiment, F_0.114_-Sap and F_0.188_-Sap reduced both crystallization time and crystal size but increased nucleation density of lysozyme crystals formed at pH 4.7, whereas F_0_-Sap showed no such effect (Table 2). F_0.114_-Sap and F_0.188_-Sap increased the relative nucleation densities with increasing silicate content in the range of 0.0 to 0.4% (Fig. 3A). Thus, these observations demonstrate F_0.114_-Sap and F_0.188_-Sap induce heterogeneous nucleation of the lysozyme crystal, and that the substitution of hydroxyl groups for fluorine atoms governs the nucleation-inducing properties of the saponite. As can be seen from the results shown in Fig. 3B, the decreases of relative nucleation density in the range of 0.4 to 0.8% are due to a decrease in supersaturation according to the adsorption of lysozyme on an excess amount of F_0.114_-Sap or F_0.188_-Sap. The morphologies of crystals in the presence of each F-Sap were the same as those of the control drops (Fig. 4A–D). Thus, the F-Saps do not appear to influence the symmetry of the lysozyme crystals.

**Table 2:** Crystallization times, crystal sizes and nucleation densities for lysozyme in the presence of F-Saps.

Layered silicate	Time	Size	Density
	(day)	(mm)	(n.c./mm^2^)
Control	2	0.41±0.16	0.67±0.32
F_0_-Sap	4	0.55±0.18	0.43±0.27
F_0.114_-Sap	1.5	0.29±0.13	2.16±0.94
F_0.188_-Sap	1.5	0.22±0.12	7.14±2.14

Crystallization conditions: starting lysozyme concentration 20 mg/mL, starting silicate content 0.2%, precipitant agent sodium chloride 1.0 M in sodium acetate 0.2 M pH 4.7. Crystallization time was defined as the time that elapses before the appearance of the first crystal. Crystal size refers to the average length of the longitudinal axis of the crystal calculated on a set of several hundreds of crystals. Nucleation density is expressed as the number of crystals per mm^2^. Reported values are mean ± SD, *n* = 6.

Data collections of lysozyme crystals formed in the absence and presence of F-Saps were carried out (Table S3). Lysozyme crystals were prepared in mother liquor containing 30% glycerol so that they could be flash cooled without soaking them in a cryoprotectant. All the crystals, regardless of the absence/presence of F-Saps, belonged to the space group P4_3_2_1_2. Indeed, we previously reported no change in the space group of thaumatin crystals formed in the presence of layered silicate [20]. In terms of quality, each completeness and average I/Sigma from the crystals formed in the presence of F-Saps was lower than those obtained from the control. F_0.188_-Sap gave the lowest completeness and average I/Sigma, and the highest mosaicity. F_0.114_-Sap gave the lowest R_merge_. Thus, unfortunately, the quality of lysozyme crystals formed in the presence of F-Saps tends to be lower than those in the absence of the nucleant. Our results are consistent with the previous data, which showed that the growth speed of thaumatin crystal formed in the presence of layered silicate is inversely related to their quality [20].

The effects of FA-Saps on lysozyme crystallization were also investigated (Fig. S6). FA-Saps did not change the nucleation density and the crystallization time by comparison with control samples, indicating that the fluoroalkyl chains binding to saponite does not induce nucleation. These results are consistent with our proposal that substitution of hydroxyl groups for fluorine atoms is essential for the promotion of lysozyme crystal nucleation.

### Crystallization of various proteins using fluoro-substituted saponites

Twelve proteins chosen to cover a wide range of pIs and molecular weights were crystallized in the presence of F-Saps (Table 3). Lysozyme was also crystallized to investigate whether the different type of precipitant affect heterogeneous nucleation.

**Table 3:** Crystallization times (days) of twelve proteins in the presence of F-Saps.

Protein				Control	F_0_-Sap	F_0.114_-Sap	F_0.188_-Sap
Sample name	MW (kDa)	pI	Charge				
Aprotinin	6.5	9.2	+	7	7	7	2
Avidin	14.3	9.7	+	5	5	5	1
Concanavalin A	25.6	6.0	+	26	26	20	13
Lysozyme	14.3	9.3	+	N. C.	N. C.	N. C.	22
Thaumatin	22.2	8.3	+	55	28	12	25
Trypsin	23	10.1	+	N. C.	42	43	34
Albumin	66	4.7	-	N. C.	26	19	14
Catalase	240	5.5	-	12	8	8	5
Glucose isomerase	172	5.0	-	N. C.	N. C.	2	2
Proteinase K	28.9	8.3	-	N. C.	N. C.	N. C.	8
*L*-Lactate dehydrogenase	32.8	5.9	-	N. C.	N. C.	8	5
Xylanase	21	9.0	±	8	9	9	2

The MW and the classical pI values were calculated using a ExPASy server tool (http://ca.expasy.org/). The charge represents the signs of net charge of each protein under each crystallization condition. ‘N. C.’ indicates that no crystal formed after two months.

As shown in Table 3, F_0.188_-Sap reduced crystallization times of six proteins compared with the control experiment, but crystallized the other six proteins at metastable conditions where spontaneous crystallization does not occur. These observations indicate that F_0.188_-Sap promotes the nucleation of all tested proteins regardless of their differences in net charge. Meanwhile, F_0.114_-Sap reduced the crystallization times of catalase, concanavalin A and thaumatin, and crystallized albumin, glucose isomerase, *L*-lactate dehydrogenase and trypsin under metastable conditions. These findings indicate that F_0.114_-Sap promotes the nucleation of seven out of the twelve test proteins. F_0_-Sap reduced the crystallization times of albumin and trypsin, and crystallized catalase and thaumatin under metastable conditions, indicating that F_0_-Sap promotes the nucleation of four out of the twelve test proteins. The addition of Izit crystal dye confirmed that no salt crystals are formed regardless of the absence/presence of F-Saps, suggesting that the silicates do not increase the production of salt crystals under the supersaturation conditions. Thus, our data suggest that the negatively charged surface of saponites with substituted fluorine atoms reduce the free energy barrier to the formation of a stable crystal nucleus of proteins with various pIs and molecular weights.

A comparison between the effects of the different saponites is shown in Table 3. F_0.114_-Sap and F_0.188_-Sap crystallized glucose isomerase and *L*-lactate dehydrogenase, whereas F_0_-Sap did not. F_0.114_-Sap and F_0.188_-Sap crystallized albumin, concanavalin A and thaumatin earlier than F_0_-Sap. These observations show that the fluoride substitution governs and enhances the nucleation potential of saponite. Additionally, only F_0.188_-Sap promoted the nucleation in the case of aprotinin, avidin, proteinase K, xylanase and lysozyme, suggesting the existence of a threshold amount of fluorine atoms required for the promotion of nucleation. Accordingly, we speculate that the optimal content of fluorine atoms in the lamellar network provides a suitable environment for the heterogeneous nucleation.

In the case of lysozyme, F_0.188_-Sap promoted nucleation in a precipitant solution containing sodium chloride (Table 2) and polyethylene glycol 4000 (Table 3), indicating that the promoting effect of F_0.188_-Sap is independent of the type of precipitant *i.e.,* salts *versus* nonionic polymers.

It is well known that porous media with a broad size distribution act as effective nucleants for crystallization of proteins [17]. In this study, all F-Saps that promoted the nucleation of crystals of albumin, catalase, thaumatin and trypsin possessed mesopores (Fig. S3), regardless of the presence/absence of fluorine atoms (Table 3). Thus, for these four proteins at least, the negatively charged trioctahedral lamellar structure with mesoporosity independently or synergistically promotes the mechanism of crystallization.

### Initial screening for crystallization using the fluoro-substituted saponite

F_0.188_-Sap, the most successful nucleant in this study, was used to search suitable initial crystallization conditions for proteins. We chose lysozyme and glucose isomerase as model proteins which are often used for testing the effect of nucleants in primary screening [32], [33]. Each protein was crystallized by the sitting-drop vapor diffusion technique using Micro-Bridges precoated with saponite. Saponite is swellable mineral which allows water to splay the silicate uniformly resulting in a desirable inorganic coat that will be tightly adhered to the bridge surface. The use of the bridge in sitting-drop screening eliminates the need for a powder-dispersing step which generally is needed for dispersing silicate particles. Therefore this method prevents bubbling on the precipitant solution providing an enhanced handling property as compared with the conventional method.

As expected, the saponite promoted the crystallization of lysozyme and glucose isomerase enhancing the success rate of protein crystallization compared to the control experiment in which no crystals were formed (Table S5). The promotion was independent of the type of precipitant, like for example salts (sodium chloride, sodium citrate tribasic dehydrate, sodium formate and ammonium sulfate), nonionic polymers (polyethylene glycol 4000, polyethylene glycol 8000 and polyethylene glycol monomethyl ether 5000) and alcohols (2-methyl-2, 4-pentandiol and 1, 6-hexanediol). Salt crystals formed regardless of the absence/presence of the saponite under some specific conditions, *e.g.* Crystal Screen Nos. 6 and 24 in lysozyme crystallization and Crystal Screen 2 No. 21 in glucose isomerase crystallization. Our screening experiments suggest that the inorganic coat promotes nucleation of protein crystals regardless of the precipitant solution, as is the case when using the silicate dispersion. In addition note that F_0.188_-Sap inhibited lysozyme crystallization in the Crystal Screen No. 40 (0.1 M sodium citrate tribasic dehydrate pH 5.6, 20% v/v 2-propanol and 20% w/v polyethylene glycol 4000) contrary to the spontaneous crystallization that occurs in the absence of the silicate (Table S5). This observation may be due to a decrease in supersaturation according to the adsorption of lysozyme on an excess amount of saponite. Our findings imply that fluorinated layered silicate can enhance the success rate in the initial screening for a large variety of proteins and reduce the time and cost of crystallization. Such crystallization method may be realized as automated system which allows for the simultaneous or sequential examination of a large number of samples. Further use of the silicate for solving the structures of new proteins will open up the potential for new screening tools in macromolecular crystallography.

### Adsorption of proteins on fluoro-substituted saponites

Previous studies described the strong correlation between heterogeneous nucleation and protein adsorption on the nucleants [27], [30]. We investigated the adsorption of protein molecules at the F-Sap/water interface under the crystallization conditions (Table 4). Below each condition, lysozyme, glucose isomerase and xylanase have a net positive, negative and neutral charge, respectively. As shown in Table 4, the adsorption densities of each protein increased in the following sequence: F_0_-Sap < F_0.114_-Sap < F_0.188_-Sap. These findings indicate that substitution results in increased protein densities at the interface. Indeed, the high protein concentration correlates with the effective promotion of heterogeneous nucleation on the silicate surface.

**Table 4:** Adsorption densities (mg/m^2^) of proteins on F-Saps.

	F_0_-Sap	F_0.114_-Sap	F_0.188_-Sap
Lysozyme	2.30±0.03	2.50±0.05	2.71±0.08
Glucose isomerase	0.75±0.01	0.77±0.02	0.81±0.03
Xylanase	0.84±0.01	0.87±0.01	0.90±0.02

**Figure 5:**
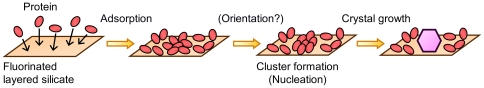
Proposed model for heterogeneous nucleation of protein crystal on fluorinated layered silicate.

Several reports identify a relationship between the density of protein molecules adsorbed on negatively charged surfaces, such as layered silicate or silica, and the orientation of the molecules [38], [39], [43]. We propose a model of heterogeneous nucleation on layered silicate that is associated with this orientation (Fig. 5). At the initial stage of the nucleation, local interactions between the negatively charged hydroxyl groups or fluorine atoms of the silicate and positively charged residues of the protein molecule will occur sequentially. In the case of the silicate without fluorine atoms, the neighboring protein-protein interactions at the interface are likely to be minor because of the relatively large intermolecular distances. Therefore the adsorbed molecules assume a relatively wide distribution of orientations. By contrast, in the case of fluorinated layered silicates with substituted fluorine atoms, lateral electrostatic repulsions of neighboring protein molecules will spontaneously force them into a narrow distribution of orientations. Hence, the positively charged patches on the protein molecules will be positioned close to the surface to minimize repulsion. The high local supersaturation of adsorbed molecules with a narrow distribution of orientations results in the formation of a critical cluster comprised of neighboring molecules with suitable bond angles on the silicate surface. The cluster acts as a nucleus, leading to crystal growth in the supersaturation conditions. Additional studies for investigating the adsorbed proteins will help to understand the molecular mechanism of heterogeneous nucleation.

In conclusion, fluorinated layered silicate promotes nucleation of crystals of a wide range of proteins. We believe that application of the silicate for screening will have a major impact in structural biology.

## Materials and Methods

### Materials

Saponite was kindly donated by Kunimine Industries Co., Ltd. (Tokyo, Japan). Montmorillonite was kindly donated by Dr. Takeo Ebina (National Institute of Advanced Industrial Science and Technology). Fluorohectorite, hectorite, K-tetrasilicic fluoromica and Na-tetrasilicic fluoromica were obtained from CO-OP Chemical Co., Ltd. (Tokyo, Japan). Sericite and pyrophyllite were obtained from The Clay Science Society of Japan. Fluorophlogopite was obtained from Topy Industries Co., Ltd. (Tokyo, Japan) and Muscovite from Yamaguchi Mica Co., Ltd. (Aichi, Japan). Talc was purchased from Nippon Talc Co., Ltd. (Osaka, Japan). Hen egg-white lysozyme, bovine liver catalase, proteinase K from *Tritirachium album* and bovine pancreas trypsin were obtained from Wako Pure Chemical Industries, Ltd. (Osaka, Japan). Aprotinin, jack bean concanavalin A, thaumatin from *Thaumatococcus daniellii*, human serum albumin, Sigmacote and 24-well tray were obtained from Sigma-Aldrich (St Louis, MO). Glucose isomerase from *Streptomyces rubiginosus*, xylanase from *Trichoderma longibrachiatum*, Micro-Bridge, Crystal Screen, Crystal Screen 2, and Izit crystal dye were obtained from Hampton Research (Aliso Viejo, CA). Avidin from egg white was purchased from Nacalai Tesque, Inc. (Kyoto, Japan). *L*-Lactate dehydrogenase from *Thermus caldophilus* GK24 was expressed and purified as described previously [44]. Coverslips were obtained from Matsunami Glass Ind., Ltd. (Osaka, Japan). High vacuum grease was obtained from Dow Corning Toray Co., Ltd. (Tokyo, Japan). Perfluorooctyltriethoxysilane was obtained from Tokyo Kasei Co., Ltd. (Japan). All other chemicals were obtained from Nacalai Tesque, Inc., Wako Pure Chemical Industries, Ltd. and Sigma-Aldrich.

### Synthesis of fluorinated saponites

Fluoro-substituted saponites (F-Saps) were synthesized from gels under hydrothermal conditions as described previously [35], [36], [45]–[48]. The chemical composition of the gel phase was chosen to correspond to the theoretical composition of saponite Na_0.33_(Mg_3_)(Al_0.33_Si_3.67_)O_10_(OH, F)_2_. The gels were prepared using water glass as the silicon source, MgSO_4_, Al_2_(SO_4_)_3_ and NaF. The mixture was hydrothermally treated in 3.0 L pressure vessels and autoclaved at 250°C for 4 h under autogenous pressure. After cooling the vessel, the solid product was washed twice in deionized water to remove any unreacted NaF and then dried overnight at 105°C. Finally, the product was ground and the resulting silicate powder used for protein crystallization. As the control material, saponite without fluoride substitution was synthesized using NaOH instead of NaF.

Fluoro-alkylated saponites (FA-Saps) were also synthesized. The silane solution was prepared by mixing 0.15–1.2 g of perfluorooctyltriethoxysilane and 240 mL of 90% methanol, and then 1.5 g of saponite was dipped into the solution while stirring to give the coverage of 9.6–76.5%. After stirring for 1 h at 60°C, the solid phase was washed twice in deionized water and dried for 24 h at 60°C.

### Characterization

Fluorine identification was carried out using a scanning electron microscope (Hitachi S-800) equipped with an energy dispersive X-ray (SEM-EDX) spectrometer (KEVEX Delta series) at an operating voltage of 10 kV. Chemical composition was determined using X-ray photoelectron spectroscopy (XPS) (Rigaku RIX1000). Nitrogen adsorption/desorption at liquid nitrogen temperature were measured using an AUTOSORB-1 (Quantachrome instruments, Boynton Beach, FL). Prior to the measurement, each silicate was degassed for 3 h at 473 K under vacuum. Specific surface area was determined from the adsorption data ranging from relative pressure P/P_0_ = 0.05–0.30 according to the Brunauer–Emmett–Teller (BET) method. Total pore volume was estimated from the amount of nitrogen adsorption at the maximum relative pressure. Pore distribution was calculated from the adsorption data using the Barrett–Joyner–Halenda (BJH) method. Cation exchange capacity (CEC) was determined by the ammonium acetate method as described previously [49]. Zeta potential was measured with an ELS-Z instrument (Otsuka Electronics Co. Ltd., Tokyo, Japan). Hydrophobicity was determined as described previously [50]. Fourier transform infrared (FT-IR) spectra of FA-Saps were obtained on a Spectrum 100 FTIR spectrometer (Perkin Elmer, Boston, MA).

### Crystallization

Twelve proteins were crystallized by the hanging-drop or sitting-drop vapor diffusion technique using silicate dispersion as described previously [20]. These proteins include albumin, aprotinin, avidin, catalase, concanavalin A, glucose isomerase, *L*-lactate dehydrogenase, lysozyme, proteinase K, thaumatin, trypsin and xylanase. Crystallization conditions of proteins used are listed in Table S4. For setting up the crystallization experiments, 5 µL of each protein solution was mixed with an equal volume of a precipitant solution containing 0.1–0.8% F-Sap on a coverslip or a bridge coated with silicon. The droplet was incubated at 293±1 K in a sealed well containing a reservoir solution. We also crystallized each protein in the absence of silicate as a control. Izit crystal dye was used to distinguish whether the crystal was a protein or a salt. All experiments were carried out at least three times to test reproducibility.

Diffraction data of lysozyme crystals formed in the presence of 30% glycerol were collected with CCD camera at the BL6A station of the Photon Factory, High Energy Accelerator Research Organization (KEK). The crystals were directly flash-cooled under a stream of nitrogen gas at 100 K without soaking in a cryoprotectant. Diffraction images were indexed, integrated and then scaled using the DPS/MOSFLM program suite.

### Crystallization screening

Initial screening trials for suitable crystallization conditions of lysozyme and glucose isomerase were established by sitting-drop vapor diffusion technique using commercially available sparse-matrix screening kits: Crystal Screen or Crystal Screen 2 from Hampton Research. 5 µL of 0.2% F_0.188_-Sap dispersed in deionized water was applied over a bridge and then dried at room temperature under vacuum. The resulting bridges precoated with the saponite were used for the screening experiments. Lysozyme was dissolved in 20 mM sodium phosphate pH 7.0 to a final concentration of 8.0 mg/mL. In glucose isomerase case it was dissolved in 20 mM Tris-HCl pH 7.0 containing 1 mM MgCl_2_ to a final concentration of 13.0 mg/mL. 5 µL of each protein solution was mixed with an equal volume of the precipitant solution from the reservoir on the bridge. The droplet was incubated at 293±1 K for two months in the sealed well. We also crystallized each protein on the bridge without saponite as control. Izit crystal dye was used to distinguish whether the crystal was a protein or a salt. All experiments were carried out three times to test reproducibility.

### Protein adsorption

Batch adsorptions of lysozyme, xylanase and glucose isomerase under each crystallization condition were carried out using F-Saps. A 500 µL aliquot of each protein solution listed in Table S4 was mixed with an equal volume of precipitant solution containing 1.0% F-Sap listed in Table S4, and then stirred for 3 h at 293±1 K. After centrifugation, the concentration of supernatant was determined by the absorbance at 280 nm or Bradford assay. All experiments were carried out at least three times to test reproducibility.

## Supporting Information

Figure S1 **Schematic representation of typical layered silicate having a 2:1 type structure.**
(TIF)

Figure S2 **X-ray diffraction patterns of F0.188-Sap (A), F0.114-Sap (B) and F0-Sap (C).**
(TIF)

Figure S3 **Nitrogen adsorption/desorption isotherms of F0-Sap (A), F0.114-Sap (B) and F0.188-Sap (C).**
(TIF)

Figure S4 **Measured zeta potentials and hydrophobicities of F-Saps in the condition for lysozyme crystallization.** Reported values are mean ± SD, *n* = 3.
(TIF)

Figure S5 **Characterization of FA-Saps.** FT-IR spectra of FA-Saps with different coverage; Raw saponite (A), FA_0_-Sap (B), FA_9.6_-Sap (C), FA_19.1_-Sap (D), FA_38.2_-Sap (E), FA_57.4_-Sap (F) and FA_76.5_-Sap (G).
(TIF)

Figure S6 **Nucleation densities for lysozyme in the presence of FA-Saps with different coverage.** Crystallization conditions: starting lysozyme concentration 20 mg/mL, precipitant agent sodium chloride 1.0 M in sodium acetate 0.2 M, pH 4.7. Reported values are mean ± SD, *n* = 6.
(TIF)

Table S1 **Chemical compositions (wt%) of F-Saps obtained from XPS analysis.**
(DOC)

Table S2 **CEC, specific surface area and surface charge density of F-Saps.**
(DOC)

Table S3 **Data collection from lysozyme crystals.**
(DOC)

Table S4 **Crystallization conditions of proteins used in this study.**
(DOC)

Table S5 **Protein crystallization screening with F_0.188_-Sap in combination with the commercially available sparse-matrix screening kits.**
(DOC)
