# Supplementary figures and images for: Heterogeneous Nucleation of Protein Crystals on Fluorinated Layered Silicate
Source: PLoS One. 2011 Jul 27;6(7):e22582. doi: 10.1371/journal.pone.0022582 (PMC3144907; doi:10.1371/journal.pone.0022582)

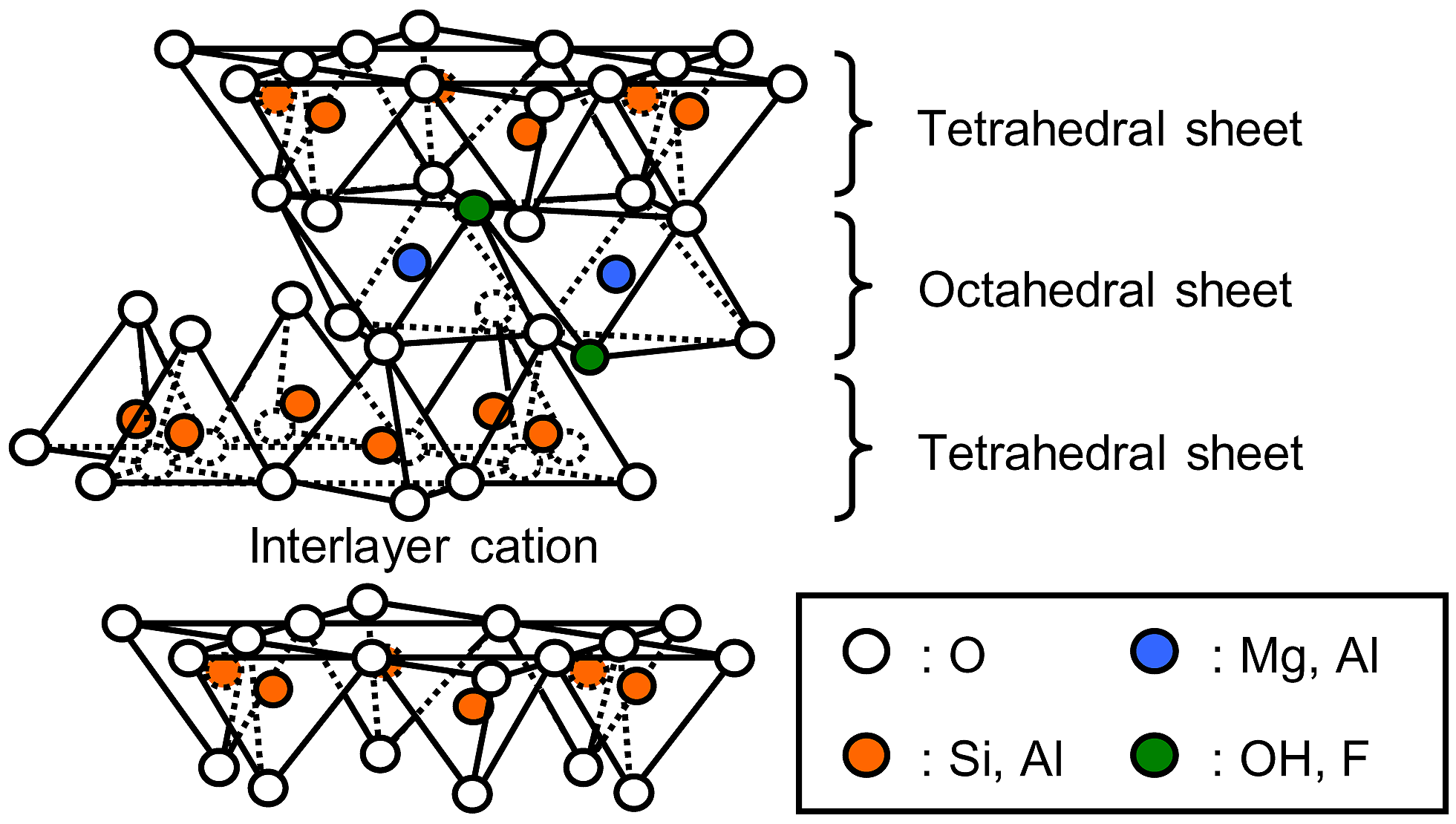

Supplement: Figure S1 — Schematic representation of typical layered silicate having a 2:1 type structure. (TIF) [file pone.0022582.s001.tif]

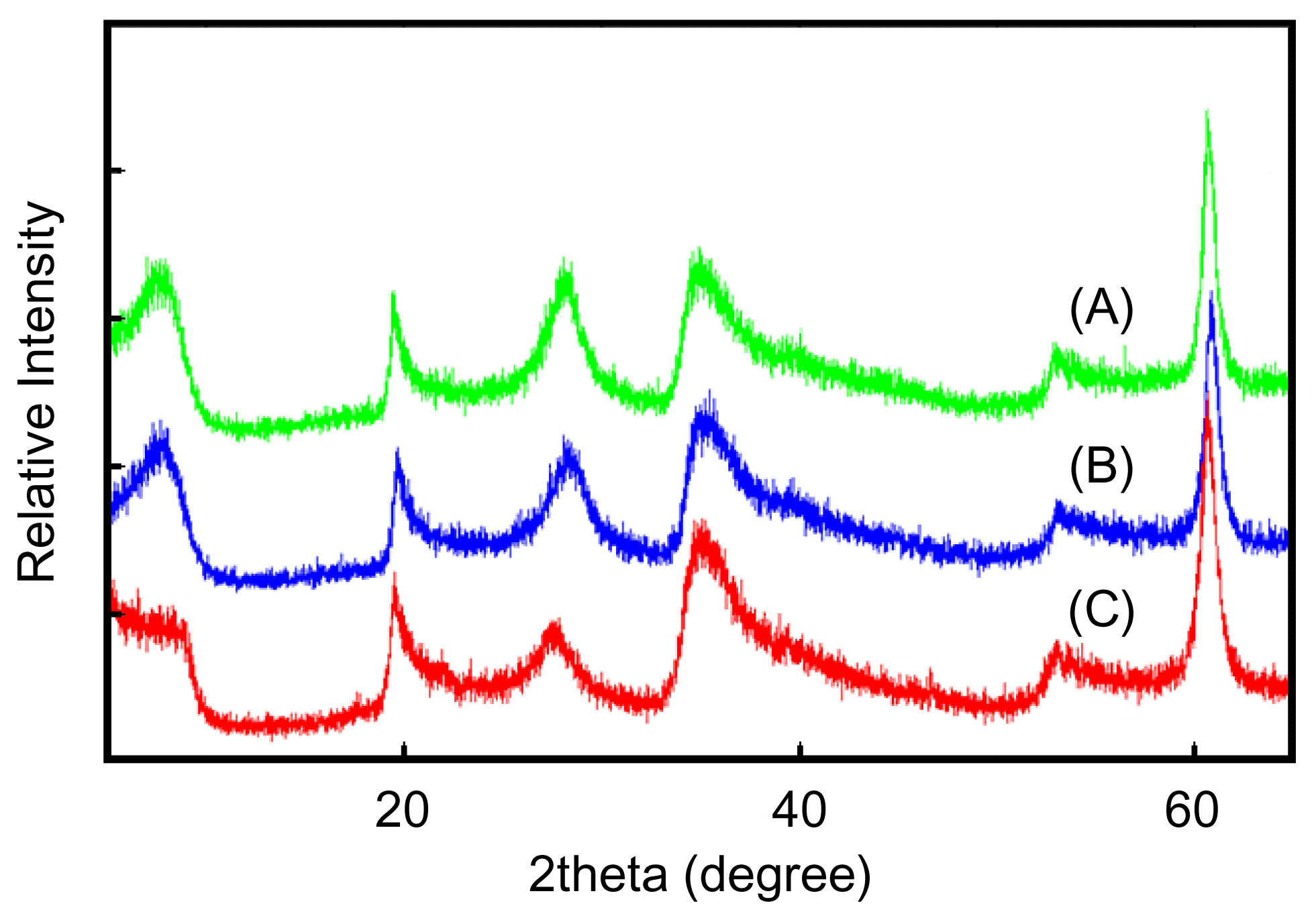

Supplement: Figure S2 — X-ray diffraction patterns of F0.188-Sap (A), F0.114-Sap (B) and F0-Sap (C). (TIF) [file pone.0022582.s002.tif]

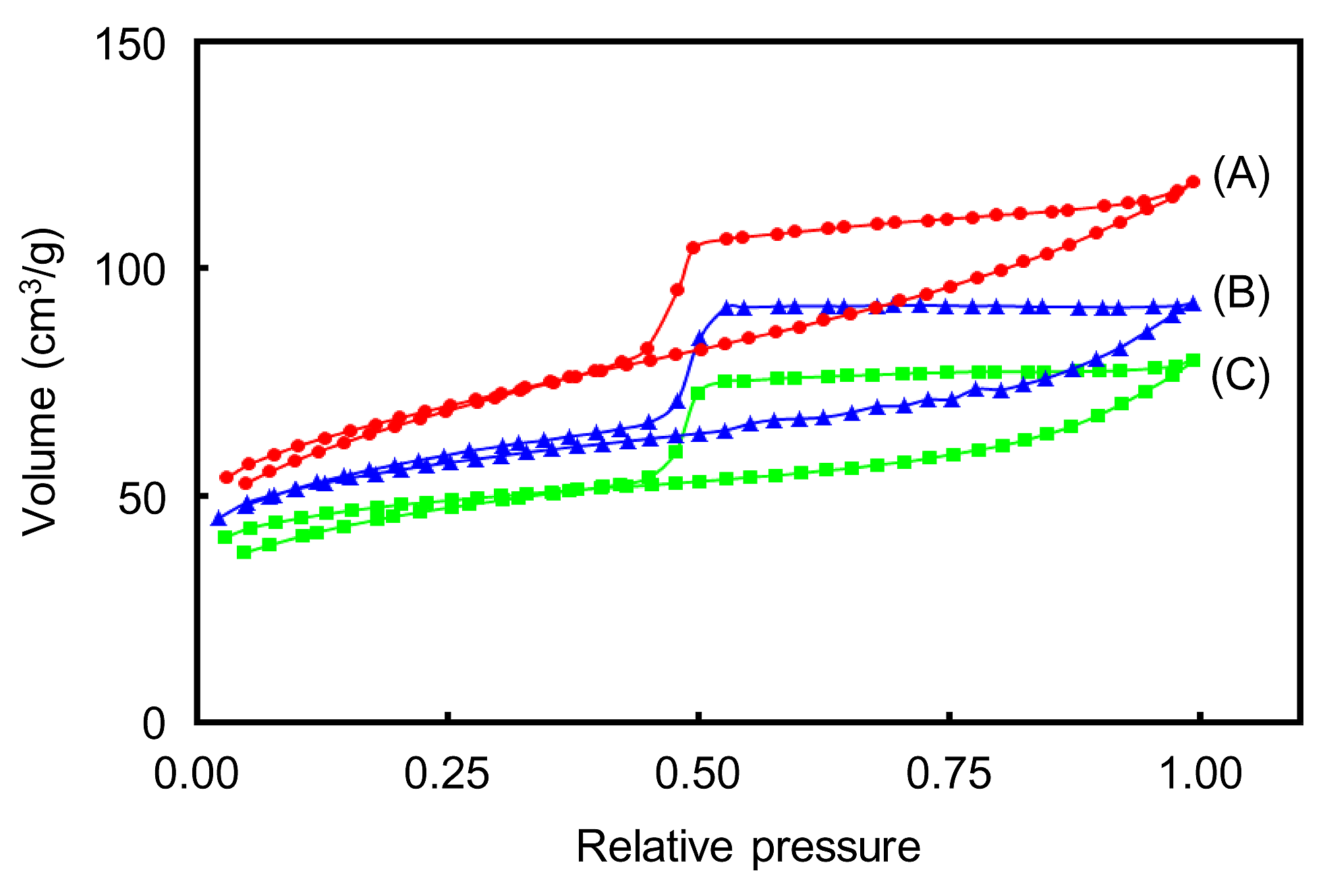

Supplement: Figure S3 — Nitrogen adsorption/desorption isotherms of F0-Sap (A), F0.114-Sap (B) and F0.188-Sap (C). (TIF) [file pone.0022582.s003.tif]

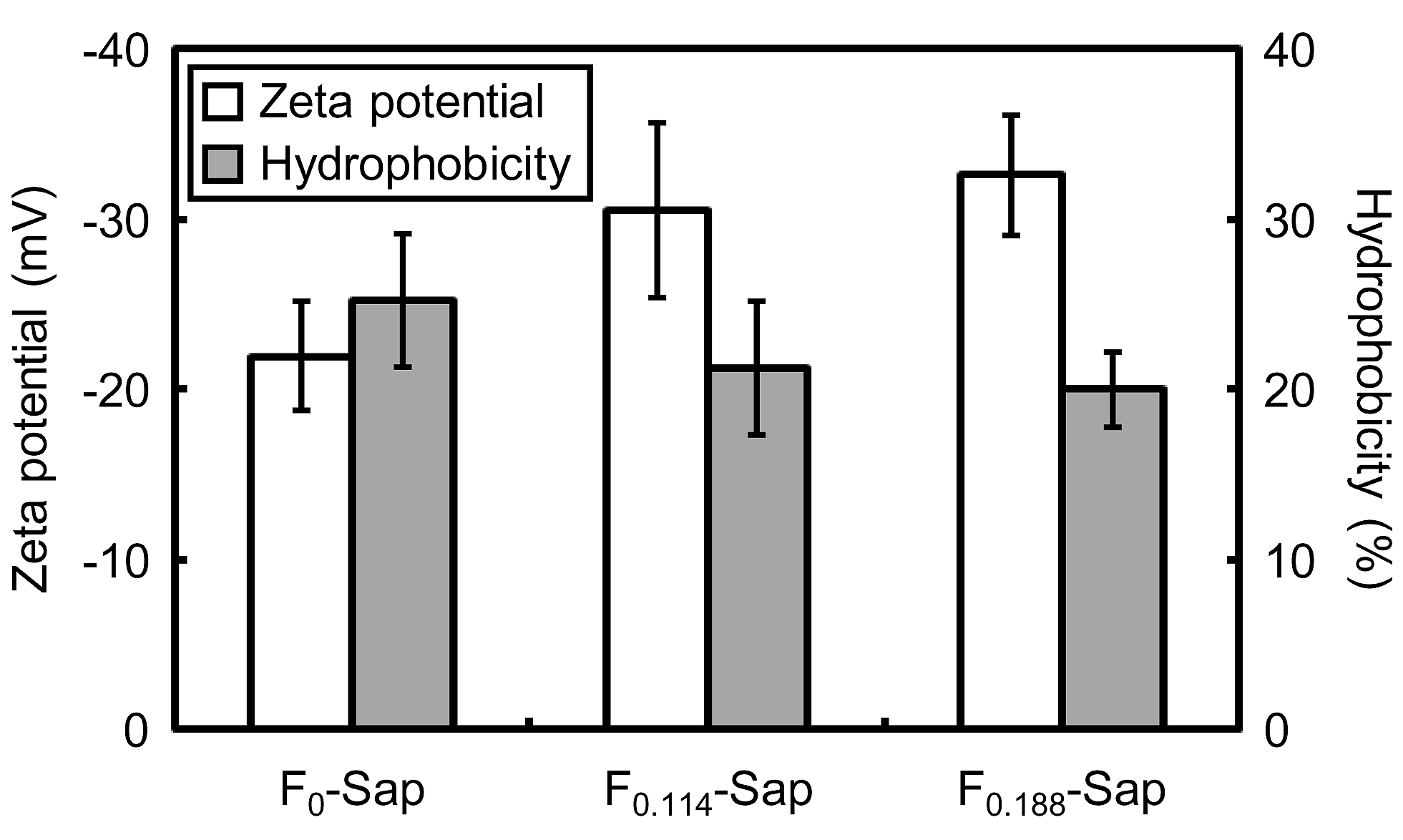

Supplement: Figure S4 — Measured zeta potentials and hydrophobicities of F-Saps in the condition for lysozyme crystallization. Reported values are mean ± SD, n = 3. (TIF) [file pone.0022582.s004.tif]

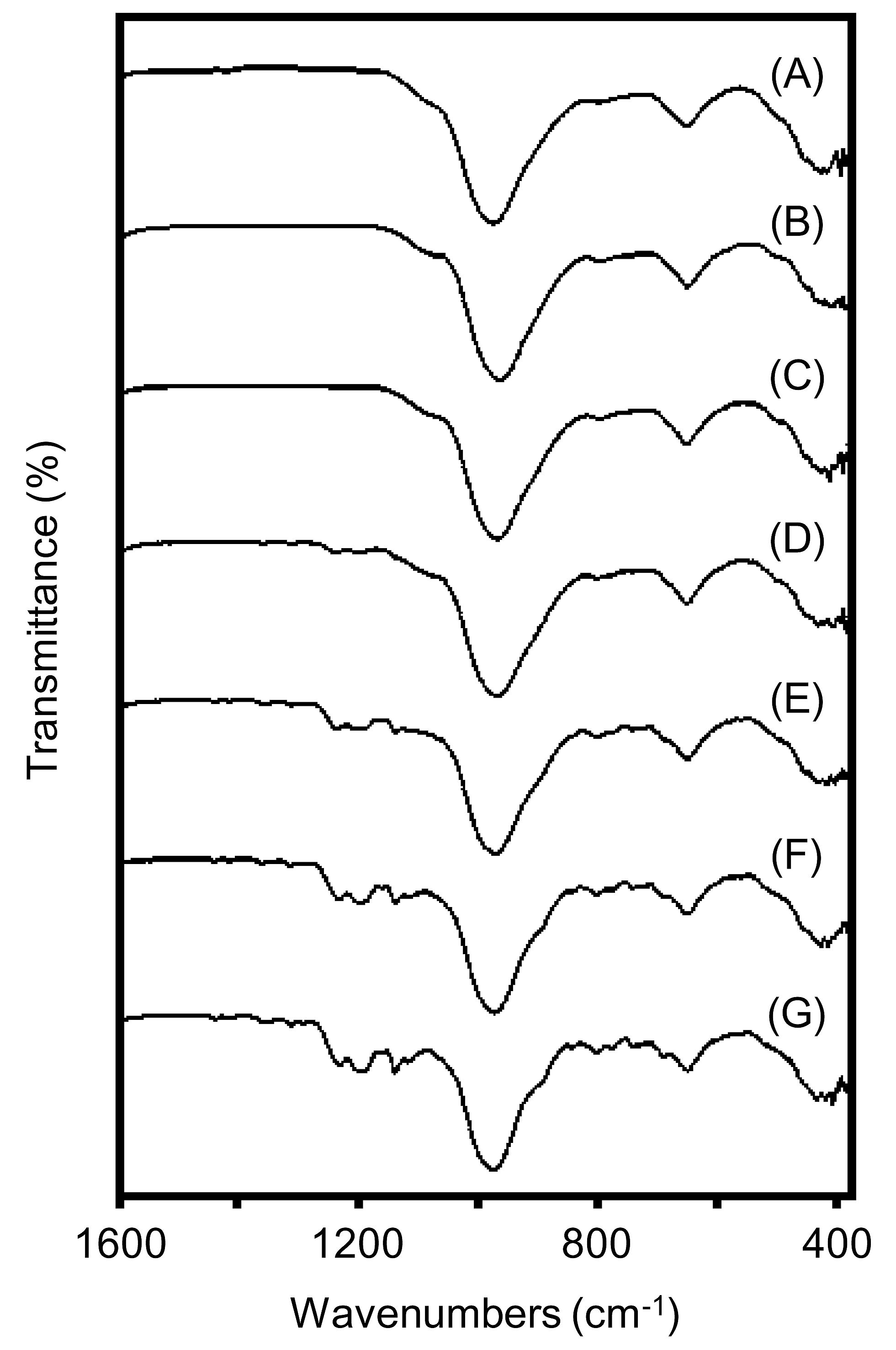

Supplement: Figure S5 — Characterization of FA-Saps. FT-IR spectra of FA-Saps with different coverage; Raw saponite (A), FA0-Sap (B), FA9.6-Sap (C), FA19.1-Sap (D), FA38.2-Sap (E), FA57.4-Sap (F) and FA76.5-Sap (G). (TIF) [file pone.0022582.s005.tif]

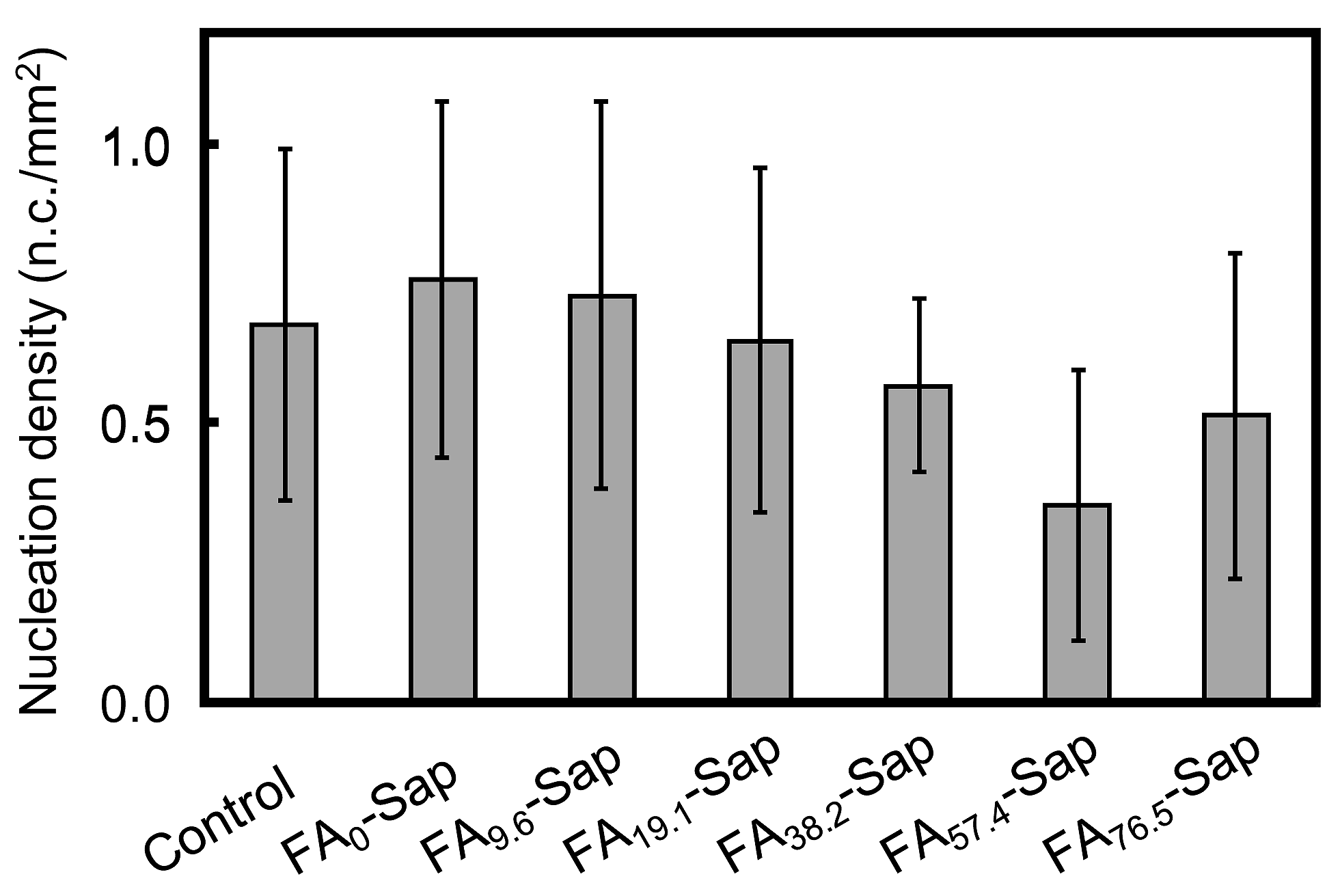

Supplement: Figure S6 — Nucleation densities for lysozyme in the presence of FA-Saps with different coverage. Crystallization conditions: starting lysozyme concentration 20 mg/mL, precipitant agent sodium chloride 1.0 M in sodium acetate 0.2 M, pH 4.7. Reported values are mean ± SD, n = 6. (TIF) [file pone.0022582.s006.tif]
