# Supplementary material for: Heterogeneous Nucleation of Protein Crystals on Fluorinated Layered Silicate
Source: PLoS One. 2011 Jul 27;6(7):e22582. doi: 10.1371/journal.pone.0022582 (PMC3144907; doi:10.1371/journal.pone.0022582)
Supplement: Table S1 — Chemical compositions (wt%) of F-Saps obtained from XPS analysis. (DOC) [file pone.0022582.s007.doc]

**Table S1.** Chemical compositions (wt%) of F-Saps obtained from XPS analysis.

|  | **F_0_-Sap** | **F_0.114_-Sap** | **F_0.188_-Sap** |
| --- | --- | --- | --- |
| SiO_2_ | 61.3 | 56.4 | 55.5 |
| Al_2_O_3_ | 5.66 | 5.41 | 5.23 |
| Fe_2_O_3_ | 0.03 | 0.01 | 0.02 |
| TiO_2_ | 0.02 | 0.01 | 0.01 |
| MgO | 26.0 | 24.9 | 24.5 |
| CaO | 0.03 | 0.05 | 0.05 |
| Na_2_O | 1.44 | 1.90 | 2.03 |
| K_2_O | 0.01 | 0.01 | 0.01 |
| CuO | 0.01 | 0.01 | 0.01 |
| Cr_2_O_3_ | 0.00 | 0.02 | 0.02 |
| SO_3_ | 0.02 | 0.01 | 0.01 |
| F | 0.00 | 0.53 | 0.86 |
| Cl | 0.01 | 0.06 | 0.04 |
| Ig-loss | 5.47 | 10.6 | 11.7 |
| Total | 100 | 99.9 | 100 |
