# Supplementary material for: Heterogeneous Nucleation of Protein Crystals on Fluorinated Layered Silicate
Source: PLoS One. 2011 Jul 27;6(7):e22582. doi: 10.1371/journal.pone.0022582 (PMC3144907; doi:10.1371/journal.pone.0022582)
Supplement: Table S2 — CEC, specific surface area and surface charge density of F-Saps. (DOC) [file pone.0022582.s008.doc]

**Table S2.** CEC, specific surface area and surface charge density of F-Saps.

| **Layered silicate** | **CEC** | **Surface area** | **Surface charge density** |
| --- | --- | --- | --- |
|  | **(meq/100 g)** | **(m^2^/g)** | **(10^-3^ meq/m^2^)** |
| F_0_-Sap | 70.6 | 227.23 | 3.11 |
| F_0.114_-Sap | 84.1 | 176.66 | 4.76 |
| F_0.188_-Sap | 94.3 | 151.45 | 6.23 |

The specific surface area was estimated using the BET theory. The surface charge density was assumed as CEC/surface area.
