# Supplementary material for: Heterogeneous Nucleation of Protein Crystals on Fluorinated Layered Silicate
Source: PLoS One. 2011 Jul 27;6(7):e22582. doi: 10.1371/journal.pone.0022582 (PMC3144907; doi:10.1371/journal.pone.0022582)
Supplement: Table S3 — Data collection from lysozyme crystals. (DOC) [file pone.0022582.s009.doc]

**Table S3.** Data collection from lysozyme crystals.

|  | **Control** | **F_0_-Sap** | **F_0.114_-Sap** | **F_0.188_-Sap** |
| --- | --- | --- | --- | --- |
| Temperature of crystallization (K) | 293 | 293 | 293 | 293 |
| Temperature of data collection (K) | 100 | 100 | 100 | 100 |
| Space group | P4_3_2_1_2 | P4_3_2_1_2 | P4_3_2_1_2 | P4_3_2_1_2 |
| Unit-cell parameters (Å) | a=b=78.498, c=36.913 | a=b=78.594, c=36.908 | a=b=78.647, c=36.903 | a=b=78.365, c=36.929 |
| Wavelength (Å) | 0.97800 | 0.97800 | 0.97800 | 0.97800 |
| Resolution (Å) | 50.00-1.19 | 50.00-1.19 | 50.00-1.19 | 50.00-1.19 |
| Total number of reflections | 475498 | 370989 | 234129 | 238588 |
| Number of unique reflections | 37625 | 37660 | 37716 | 37470 |
| Mosaicity range | 0.38-0.53 | 0.38-0.66 | 0.15-0.17 | 0.75-1.29 |
| Completeness (%) | 97.8 | 96.0 | 97.3 | 92.2 |
| R_merge_ | 0.053 | 0.058 | 0.044 | 0.069 |
| Average I/ Sigma (I) | 37.236 | 33.651 | 33.678 | 21.126 |
