# Supplementary material for: Heterogeneous Nucleation of Protein Crystals on Fluorinated Layered Silicate
Source: PLoS One. 2011 Jul 27;6(7):e22582. doi: 10.1371/journal.pone.0022582 (PMC3144907; doi:10.1371/journal.pone.0022582)
Supplement: Table S4 — Crystallization conditions of proteins used in this study. (DOC) [file pone.0022582.s010.doc]

**Table S4.** Crystallization conditions of proteins used in this study.

| **Protein** | **Droplet condition (a 1:1 mixture)** | | **Reservoir solution** |
| --- | --- | --- | --- |
|  | **Starting protein solution** | **Starting precipitant buffer** |  |
| Lysozyme (for Table 1, 2, 4) | 20 mg/mL protein, 20 mM sodium acetate pH 4.7 | 200 mM sodium acetate pH 4.7, 1.0 M NaCl | 200 mM sodium acetate pH 4.7, 1.0 M NaCl |
| Lysozyme (for Table 3) | 8 mg/mL protein, 20 mM sodium phosphate pH 7.0 | 100 mM sodium acetate pH 4.6, 200 mM (NH_4_)_2_SO_4_, 25% PEG 4000 | 100 mM sodium acetate pH 4.6, 200 mM (NH_4_)_2_SO_4_, 25% PEG 4000 |
| Aprotinin | 28 mg/mL protein, 50 mM sodium acetate pH 4.5 | 50 mM sodium acetate pH 4.5, 2.8 M NaCl | 50 mM sodium acetate pH 4.5, 1.9 M NaCl |
| Avidin | 9.0 mg/mL protein, 50 mM sodium phosphate pH 5.7, 0.75 mM biotin | 50 mM sodium phosphate pH 5.7, 3.0 M (NH_4_)_2_SO_4_ | 50 mM sodium phosphate pH 5.7, 2.75 M (NH_4_)_2_SO_4_ |
| Concanavalin A | 8.0 mg/mL protein, 20 mM sodium citrate pH 5.8 | 200 mM sodium citrate pH 5.8, 1.0 M (NH_4_)_2_SO_4_ | 200 mM sodium citrate pH 5.8, 1.0 M (NH_4_)_2_SO_4_ |
| Thaumatin | 5.0 mg/mL protein, 20 mM PIPES pH 6.8 | 200 mM PIPES pH 6.8, 600 mM sodium potassium tartarate | 200 mM PIPES pH 6.8, 600 mM sodium potassium tartarate |
| Trypsin | 15 mg/mL protein, 25 mM HEPES pH 7.0, 10 mM CaCl_2_, 2.5 mg/mL Benzamidine | 25 mM HEPES pH 7.0, 200 mM (NH_4_)_2_SO_4_, 20% PEG 8000 | 25 mM HEPES pH 7.0, 200 mM (NH_4_)_2_SO_4_, 20% PEG 8000 |
| Albumin | 100 mg/mL protein, 50 mM potassium phosphate pH 5.0 | 50 mM potassium phosphate pH 5.0, 5 mM sodium azide, 40% PEG 400 | 50 mM potassium phosphate pH 5.0, 5 mM sodium azide, 40% PEG 400 |
| Catalase | 15 mg/mL protein, 20 mM Tris-HCl pH 8.5 | 100 mM Tris-HCl pH 8.5, 8.0% PEG 4000, 300 mM LiCl | 100 mM Tris-HCl pH 8.5, 8.0% PEG 4000, 300 mM LiCl |
| Glucose isomerase | 15 mg/mL protein, 20 mM Tris-HCl pH 7.0, 1 mM MgCl_2_ | 100 mM Tris-HCl pH 7.0, 200 mM MgCl_2_, 15% MPD | 100 mM Tris-HCl pH 7.0, 200 mM MgCl_2_,  15% MPD |
| Proteinase K | 10 mg/mL protein, 25 mM HEPES pH 7.0, 2 mM PMSF | 100 mM Tris-HCl pH 8.5, 800 mM (NH_4_)_2_SO_4_ | 100 mM Tris-HCl pH 8.5, 800 mM (NH_4_)_2_SO_4_ |
| *L*-Lactate dehydrogenase | 16 mg/mL protein, 5.0 mM MOPS pH 7.2, 50 mM NaCl | 50 mM MOPS pH 7.0, 6.0% PEG 6000 | 50 mM MOPS pH 7.0, 6.0% PEG 6000 |
| Xylanase | 15 mg/mL protein, 10 mM Bicine pH 9.0, 1.0 mM MgSO_4_, 1.0 mM DTT | 50 mM Bicine pH 9.0, 250 mM (NH_4_)_2_SO_4_, 200 mM NaI | 100 mM Bicine pH 9.0, 450 mM (NH_4_)_2_SO_4_ |
