# Supplementary material for: Heterogeneous Nucleation of Protein Crystals on Fluorinated Layered Silicate
Source: PLoS One. 2011 Jul 27;6(7):e22582. doi: 10.1371/journal.pone.0022582 (PMC3144907; doi:10.1371/journal.pone.0022582)
Supplement: Table S5 — Protein crystallization screening with F0.188-Sap in combination with the commercially available sparse-matrix screening kits. (DOC) [file pone.0022582.s011.doc]

**Table S5.** Protein crystallization screening with F_0.188_-Sap in combination with the commercially available sparse-matrix screening kits.

| **Protein** | **Screening kit** | **Layered silicate** | **Crystallization condition Nos. with crystals** | **Success rate of crystallization** |
| --- | --- | --- | --- | --- |
| Lysozyme | Crystal Screen | Control | 34, 35, 40 | 3/50 = 6.0% |
|  |  | F_0.188_-Sap | 9, 15, 17, 34, 35, 45 | 6/50 = 12.0% |
| Glucose isomerase | Crystal Screen 2 | Control | 23, 25, 26, 28, 32, 39, 43 | 7/48 = 14.6% |
|  |  | F_0.188_-Sap | 23, 25, 26, 28, 32, 35, 39, 43 | 8/48 = 16.7% |
